# Radiomic signatures with contrast-enhanced magnetic resonance imaging for the assessment of breast cancer receptor status and molecular subtypes: initial results

**DOI:** 10.1186/s13058-019-1187-z

**Published:** 2019-09-12

**Authors:** Doris Leithner, Joao V. Horvat, Maria Adele Marino, Blanca Bernard-Davila, Maxine S. Jochelson, R. Elena Ochoa-Albiztegui, Danny F. Martinez, Elizabeth A. Morris, Sunitha Thakur, Katja Pinker

**Affiliations:** 1 Department of Radiology, Breast Imaging Service, Memorial Sloan Kettering Cancer Center, 300 E 66th St, 7th Floor, New York, NY 10065 USA; 2 Department of Diagnostic and Interventional Radiology, University Hospital Frankfurt, Frankfurt, Germany; 3 Department of Biomedical Sciences and Morphologic and Functional Imaging, University of Messina, Messina, Italy; 4 Department of Epidemiology and Biostatistics, Memorial Sloan Kettering Cancer Center, New York, NY USA; 5 Department of Medical Physics, Memorial Sloan Kettering Cancer Center, New York, NY USA; 6 Department of Biomedical Imaging and Image-guided Therapy, Division of Molecular and Gender Imaging, Medical University Vienna, Vienna, Austria

**Keywords:** Radiomics, Contrast-enhanced, Magnetic resonance imaging, Breast cancer, Molecular subtype

## Abstract

**Background:**

To evaluate the diagnostic performance of radiomic signatures extracted from contrast-enhanced magnetic resonance imaging (CE-MRI) for the assessment of breast cancer receptor status and molecular subtypes.

**Methods:**

One hundred and forty-three patients with biopsy-proven breast cancer who underwent CE-MRI at 3 T were included in this IRB-approved HIPAA-compliant retrospective study. The training dataset comprised 91 patients (luminal A, *n* = 49; luminal B, *n* = 8; HER2-enriched, *n* = 11; triple negative, *n* = 23), while the validation dataset comprised 52 patients from a second institution (luminal A, *n* = 17; luminal B, *n* = 17; triple negative, *n* = 18). Radiomic analysis of manually segmented tumors included calculation of features derived from the first-order histogram (HIS), co-occurrence matrix (COM), run-length matrix (RLM), absolute gradient (GRA), autoregressive model (ARM), discrete Haar wavelet transform (WAV), and lesion geometry (GEO). Fisher, probability of error and average correlation (POE + ACC), and mutual information coefficients were used for feature selection. Linear discriminant analysis followed by *k*-nearest neighbor classification (with leave-one-out cross-validation) was used for pairwise radiomic-based separation of receptor status and molecular subtypes. Histopathology served as the standard of reference.

**Results:**

In the training dataset, radiomic signatures yielded the following accuracies > 80%: luminal B vs. luminal A, 84.2% (mainly based on COM features); luminal B vs. triple negative, 83.9% (mainly based on GEO features); luminal B vs. all others, 89% (mainly based on COM features); and HER2-enriched vs. all others, 81.3% (mainly based on COM features). Radiomic signatures were successfully validated in the separate validation dataset for luminal A vs. luminal B (79.4%) and luminal B vs. triple negative (77.1%).

**Conclusions:**

In this preliminary study, radiomic signatures with CE-MRI enable the assessment of breast cancer receptor status and molecular subtypes with high diagnostic accuracy. These results need to be confirmed in future larger studies.

**Electronic supplementary material:**

The online version of this article (10.1186/s13058-019-1187-z) contains supplementary material, which is available to authorized users.

## Background

Breast cancer is a heterogeneous disease with varying clinical presentations, subtypes, and treatment responses [1–7]. Although traditional prognostic and predictive factors such as tumor size, grade, histopathologic type, and immunohistochemical receptor status are well established, it has become evident that these traditional classifications cannot fully capture the heterogeneity of breast cancer so that the classical approach of stratifying patients into treatment groups based on phenotypic biomarkers may be insufficient in some patients. In this era of precision medicine, treatments are selected based on genetic tumor characteristics. In breast cancer, gene expression profiling has revealed four main intrinsic molecular subtypes that show pervasive differences in their gene expression patterns: luminal A, luminal B, human epidermal growth factor receptor 2 (HER2)-enriched, and triple negative [8–10]. These intrinsic molecular subtypes have different phenotypic presentations, prognosis, treatment responses, and recurrence-free and disease-specific survival leading to molecular subtype-based recommendations for systemic therapy [1–3, 11, 12].

Assessment of molecular subtypes is currently based on either gene expression profiling or immunohistochemical (IHC) surrogates from invasive tissue sampling [10, 13, 14]. It must be noted that this approach has limitations. A single biopsy of a potentially heterogenous tumor can capture only a snapshot of the tumor tissue that is subject to selection bias and may not be completely representative of the genetic, epigenetic, and/or phenotypic alterations of the entire tumor. In addition, cancer biology is subject to change over time as well with treatment from a stem-like, therapy-resistant and a differentiated drug-sensitive phenotype in a process linked to epithelial-mesenchymal transition [15]. Therefore, there is a strong argument for the development of alternative means to derive tumor characteristics that are prognostic indicators, i.e., receptor status and molecular subtypes, from the tumor in its entirety and to spatio-longitudinal monitor tumor biology changes during treatment. This is a unique opportunity for medical imaging, and in this context, contrast-enhanced magnetic resonance imaging (CE-MRI) coupled with radiomic analyses has yielded initial encouraging results. Prior studies have investigated radiomic signatures in the breast, but the generalization of these results is limited due to utilization of different MRI protocols and scanners [11, 16–18], results may be suboptimal due to inclusion of only a few radiomic features [11, 17–20], and only certain subgroups of breast cancers have been investigated [21].

We hypothesized that through the extraction of radiomic signatures from standardized CE-MRI data, an accurate assessment of molecular subtypes and receptor status of breast cancers will be provided. Thus, the aim of our study was to evaluate the diagnostic performance of CE-MRI coupled with radiomic analysis for the differentiation of breast cancers of different receptor status and molecular subtype.

## Methods

This retrospective study was compliant with Health Insurance Portability and Accountability Act guidelines and approved by the Institutional Review Board with a waiver of written informed consent.

### Patients

A prospectively populated research database was searched for patients who underwent state-of-the-art multiparametric MRI of the breast at our institution between January 2011 and January 2013 and who fulfilled the following inclusion criteria: histopathologically verified breast cancer, lesions ≥ 1 cm on CE-MRI to reduce the influence of partial volume effect on texture analysis [22]; 18 years or older; not pregnant; and not breastfeeding. One hundred and seventeen consecutive patients matched our search criteria. These patients underwent MRI for pre-treatment staging of their biopsy-proven breast cancer. Twenty-six were excluded for pathology results demonstrating types of cancer other than invasive ductal carcinoma or invasive lobular carcinoma, prior treatment, and poor image quality. Thus, 91 patients were included in the study.

### MR imaging

All patients were examined with a 3 T system (Discovery MR750; GE Healthcare, Milwaukee, WI) with the body coil as the transmitter and a dedicated phased-array breast coil as the receiver. All patients underwent a state-of-the-art MRI protocol [23] including T2-weighted imaging, diffusion-weighted imaging, and CE-MRI. CE-MRI data was used for radiomic analysis.

CE-MRI (3D T1-weighted gradient echo VIBRANT sequence; TR/TE 4.3/2.1 ms; flip angle 10^o^; matrix size 320 × 192; FOV 30 cm; voxel size 1.8–2 mm^3^) with and without fat suppression. CE-MRI images were acquired before and at three points at 60-s intervals after the injection of a standard dose (0.1 mmol/kg body weight) of gadopentetate dimeglumine (Magnevist; Bayer HealthCare, Hanover, NJ, USA). Additional axial CE-MRI images (voxel size 0.7 mm^3^, acquisition time 1.5 min) were also collected. Contrast agent was injected in an antecubital vein as a bolus using a power injector at 4 m/s, followed by a saline flush. Parallel imaging using the array spatial sensitivity encoding technique (ASSET) was applied during the acquisition of DW and CE-MR images. The details of the full MRI sequence protocol are provided in Additional file 1.

### Radiomic analysis

**Fig. 1:**
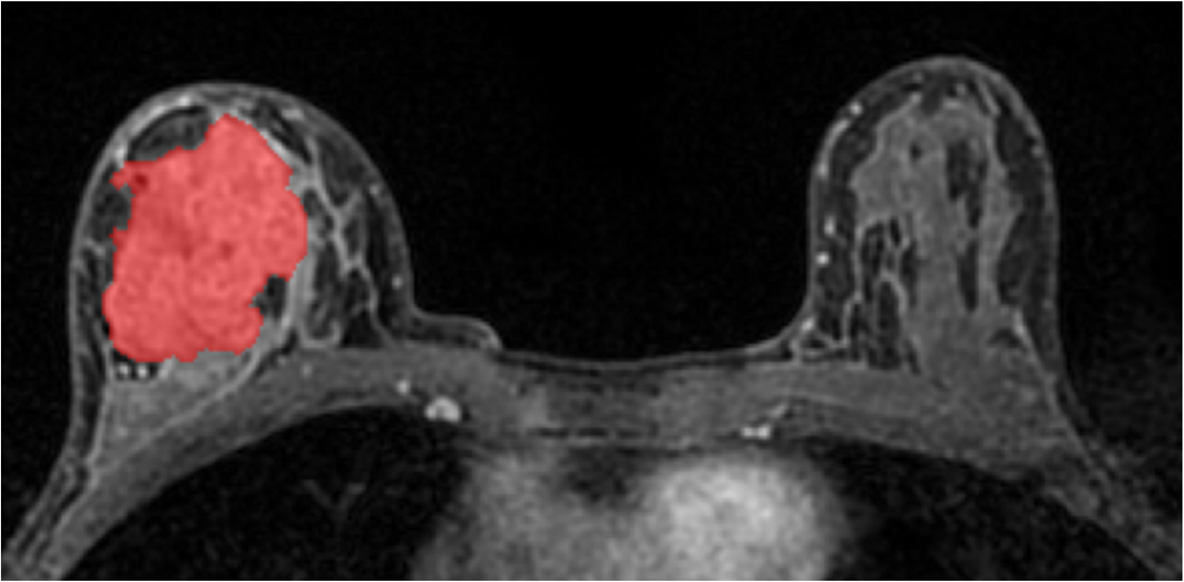
Manual region of interest placement for radiomic analysis in a 56-year-old patient with a HER2-enriched invasive ductal carcinoma in the right breast

**Fig. 2:**
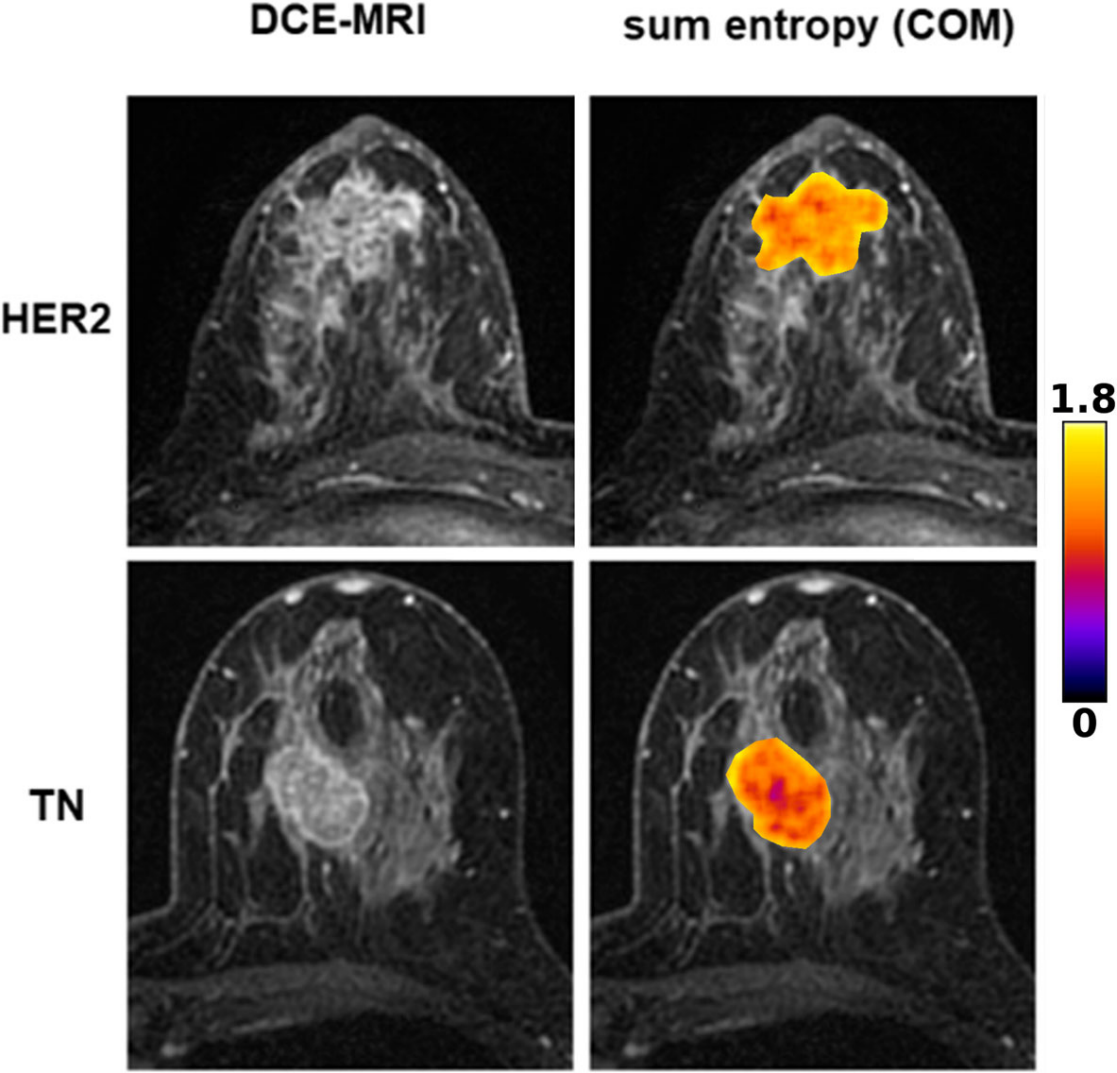
Original CE-MRI images and corresponding color-coded sum entropy feature map as overlay of the tumor area of triple-negative (TN) and HER2-enriched (HER2) breast cancer. TN shows a clearly lower sum entropy than HER2

For radiomic analysis, the first post-contrast T1-weighted sequence was used. T1 was chosen over T2 and T3 for radiomic analysis, as at this time point, malignant lesions show peak enhancement, and tumor heterogeneity/subtle lesion morphologic features are best discernable on visual assessment. A semi-automatic approach for feature extraction was chosen, using the publicly available software package MaZda 4.6 (http://www.eletel.p.lodz.pl/programy/mazda/), which was developed within the COST (European Cooperation in The Field of Scientific and Technical Research) projects B11 and B21. In all patients, the slice with the largest transaxial lesion diameter on CE-MRI was chosen and two radiologists with 13 and 4 years of experience in consensus drew a single two-dimensional region of interest around the whole tumor (Fig. 1). Adequate distance was kept from the surrounding anatomic structures and biopsy markers. Gray-level normalization of each region of interest was performed, limiting the dynamics to *μ* ± 3*σ* (μ, gray-level mean; *σ*, gray-level standard deviation) in order to reduce the influence of contrast and brightness variations which might otherwise affect radiomic feature quantification [24]. Radiomic analysis of the manually segmented lesions included calculation of features derived from first-order histogram (HIS), co-occurrence matrix (COM), run-length matrix (RLM), absolute gradient (GRA), autoregressive model (ARM), discrete Haar wavelet transform (WAV), and lesion geometry (GEO) (Fig. 2) [25–27]. All features that MaZda was capable of calculating were included. The numbers of calculated features per feature class are as follows: HIS, *n* = 9; COM, *n* = 220; RLM, *n* = 20; GRA, *n* = 5; ARM, *n* = 5; WAV, *n* = 20; GEO, *n* = 73 (total number of features per lesion, *n* = 352). The full list of radiomic features used in this study (including their abbreviations) can be accessed at http://www.eletel.p.lodz.pl/programy/mazda/download/FeaturerList.pdf. For instance, COM features are based on pairs of pixels/voxels and, hence, provide information on lesion heterogeneity. RLM parameters are calculated for four directions and represent the number of times there is a run of pixels having a certain gray level. Meanwhile, ARM is based on a local interaction between pixels, where the intensity of one pixel is assumed to be a weighted sum of neighboring intensities. In contrast to all other feature groups, HIS features represent statistical descriptors of lesion signal intensities. The total time for image post-processing including lesion segmentation and radiomic analysis was approximately 2–3 min per lesion.

### Statistical analysis

Feature selection is a method to reduce the large number of features obtained to the most relevant parameter set and is based on the choice of certain features according to given mathematical criteria. In this study, Fisher (ratio of between-class to within-class variance), minimization of probability of classification error and average correlation (POE + ACC), and mutual information coefficients were used for feature selection. Fisher did not take correlation between features into account, while the other two selection methods—POE + ACC and mutual information coefficients—however, did consider the interrelationships between features and aimed at reducing data redundancy. Feature selection was performed once across the entire training set, prior to linear discriminant analysis (LDA) and cross-validation. LDA was performed separately for each comparison to reduce the number of features used to distinguish between two subtypes/receptor status. LDA followed by *k*-nearest neighbor classification (with leave-one-out cross-validation) was used for pairwise radiomic-based separation of subtypes/receptor status, i.e., the model was trained with the use of all patients (*n*) − 1, excluding information from the held-out patient, and tested on the remaining patient. The training-testing process was then repeated *n* times, with *n* depending on the number of samples included in each comparison (e.g., luminal A vs. luminal B). LDA produces new feature vectors, which are called “most discriminating features” (MDF) and are optimized for maximum between-class scatter and minimum within-class scatter [28]. For a given dataset, the number of retained MDFs is equal to the number of eigenvalues exceeding 97% of the sum of all of them. The relevant equations can be accessed in the MaZda documentation on page 58, which can be accessed at http://www.eletel.p.lodz.pl/programy/mazda/download/mazda_manual.pdf. Since LDA is prone to overfitting due to “data piling,” where the feature space is large relative to the number of samples (URL: https://www.optimization-online.org/DB_FILE/2002/07/513.pdf), the number of features for each pairwise classification was dependent on the number of samples: we used one feature for every ten samples. This strategy—feature selection followed by feature reduction—was used to reduce the dimensionality of texture feature information as much as possible, as previously recommended [27]. The *k*-nearest neighbor classifier assigns a point in data space to a class to which its *k*-nearest neighbors belong [29]. Based on the MDF, the *k*-nearest neighbor classifier is used to measure the percentage of misclassified data vectors by comparing them with the true class affiliations.

For visualization of radiomic differences between breast lesions with different receptor status, feature maps were constructed which represent the distributions of single radiomic features within each image [27]. These feature maps were used solely for illustrative purposes.

### External validation cohort

To validate part of our results, some of the experiments were repeated using data from a second institution (approved by the local Institutional Review Board with a waiver of written informed consent). A research database was searched for patients who underwent state-of-the-art multiparametric MRI of the breast between January and September 2014 and who met the same inclusion criteria as described above. Fifty-two patients were included in the validation cohort. MR imaging parameters used for the validation cohort have been described previously [30]. Lesions were segmented using MaZda, and the same feature sets as selected before were applied to differentiate between molecular subtypes/receptor status. For validation, only those pairwise comparisons that yielded an accuracy of at least 80% were chosen.

### Histopathology

The tumor sampling results were reviewed for tumor histology, tumor and nuclear grade, and IHC status. A central review by reference pathologists for breast cancer is the standard in our institution. Evaluation of the IHC status included estrogen receptor, progesterone receptor, and HER2. Estrogen receptor or progesterone receptor-positive tumors (> 1% staining) were classified as hormone receptor positive. In all patients, the final histolopathological analysis from surgical tumor specimen was used as the reference standard. Tumor subtypes were classified as luminal A for hormone receptor positive and HER2 negative, luminal B for hormone receptor positive and HER2 positive, HER2-enriched for hormone receptor negative and HER2 positive, and triple negative for hormone receptor and HER2 negative [1, 11, 31]. Patients with equivocal HER2 status were evaluated using fluorescence in situ hybridization to detect gene amplification.

## Results

Mean lesion size of the 91 treatment-naïve, biopsy-proven breast cancers in 91 patients (mean age, 48 ± 9.7 years, range, 27–68 years) was 3.5 ± 2.3 cm (range, 1–16.6 cm). There were 70 mass lesions and 21 non-mass enhancing lesions. Hormone receptor positivity was observed in 57 patients (62.6%). Forty-nine breast cancers were luminal A (53.8%), eight were luminal B (8.8%), 11 were HER2-enriched (12.1%), and 23 were triple negative (25.3%).

Results of group-wise radiomic-based classifications including numbers of features are listed in Tables 1 and 2. Detailed results of all radiomic feature-based cancer classifications including all selection algorithms and respective MDFs are given in Additional file 1: Table S1. With the exception of HER2-enriched vs. luminal A (MDF = 2), the previously selected texture features were reduced to a single MDF. Selected feature sets for classifications with accuracies > 80% are given in Table 3, while feature sets for all pairwise classifications and selection algorithms are provided in Additional file 2: Table S2. Classification accuracies ≥ 80% were considered as clinically relevant and were achieved for molecular subtypes and receptor status as listed below.

**Table 1:** Results of group-wise radiomic feature-based cancer classifications for molecular breast cancer subtypes (training dataset)

	Luminal A	Luminal B	HER2-enriched	TN	All others
Luminal A	–	*84.2%* (MI; 6)	76.7% (POE; 6)	69.4% (POE; 7)	60.4% (MI; 9)
Luminal B	*84.2%* (MI; 6)	–	73.7% (Fisher; 2)	*83.9%* (Fisher; 3)	*89%* (POE; 9)
HER2-enriched	76.7% (POE; 6)	73.7% (Fisher; 2)	–	73.5% (POE; 3)	*81.3%* (MI; 9)
TN	69.4% (POE; 7)	*83.9%* (Fisher; 3)	73.5% (POE; 3)	–	73.6% (MI; 9)
All others	60.4% (MI; 9)	*89%* (POE; 9)	*81.3%* (MI; 9)	73.6% (MI; 9)	–

*HER2* human epidermal growth factor receptor 2, *MI* mutual information, *POE* probability of error and average correlation, *TN* triple negative. The feature selection algorithm and number of features used for classification are given in parentheses

**Table 2:** Results of group-wise radiomic feature-based cancer classifications for hormone receptor status (training dataset)

	HR positive	HER2 positive	HR negative	HER2 negative	Her2-enriched	TN	All others
HR positive	–	–	67% (POE; 9)	–	79.4% (Fisher; 7)	71.3% (Fisher; 8)	67% (POE; 9)
HER2 positive	–	–	–	73.6% (Fisher; 9)	–	59.5% (Fisher; 4)	73.6% (Fisher; 9)
HR negative	67% (POE; 9)	–	–	–	–	–	67% (POE; 9)
HER2 negative	–	73.6% (Fisher; 9)	–	–	–	–	73.6% (Fisher; 9)
HER2-enriched	79.4% (Fisher; 7)	–	–	–	–	–	–
TN	71.3% (Fisher; 8)	59.5% (Fisher; 4)	–	–	–	–	–
All others	67% (POE; 9)	73.6% (Fisher; 9)	67% (POE; 9)	73.6% (Fisher; 9)	–	–	–

*HER2* human epidermal growth factor receptor 2, *HR* hormone receptor, *MI* mutual information, *POE* probability of error and average correlation, *TN* triple negative. The feature selection algorithm and number of features used for classification are given in parentheses

**Table 3:** Selected feature sets for pairwise classifications with accuracies ≥ 80%

Luminal A vs. luminal B (MI)	Luminal B vs. TN (Fisher)	Luminal B vs. all others (POE)	HER2-enriched vs. all others (MI)
S(3,3)SumAverg S(2,2)SumEntrp S(5,-5)DifEntrp 45dgr_LngREmph S(2,-2)SumAverg S(0,1)Contrast	GeoW7 Variance GeoW9	S(5,5)InvDfMom Teta1 S(1,1)SumOfSqs S(1,-1)SumOfSqs S(2,2)SumAverg GrKurtosis Teta4 S(1,0)SumOfSqs S(4,4)InvDfMom	S(0,1)InvDfMom S(5,5)SumAverg S(1,1)Contrast S(2,-2)Contrast GeoE12 Vertl_ShrtREmp S(2,-2)DifEntrp S(0,2)Entropy S(4,-4)SumEntrp

*HER2* human epidermal growth factor receptor 2, *HR* hormone receptor, *MI* mutual information, *POE* probability of error and average correlation, *TN* triple negative

### Molecular breast cancer subtypes

Best results in terms of accuracies were achieved for the differentiation of luminal B cancers from other groups: luminal B vs. luminal A, 84.2% (mainly based on COM features selected by mutual information coefficients); luminal B vs. triple negative, 83.9% (mainly based on GEO features selected by Fisher coefficients); and luminal B vs. all others, 89% (mainly based on COM features selected by POE + ACC). In summary, luminal B cancers seemed to carry distinct radiomic signatures that enable their separation from breast cancers with other features (Tables 1 and 3).

The separation of HER2-enriched cancers was successful, with the following accuracy: HER2-enriched vs. all others, 81.3% (mainly based on COM features selected by mutual information coefficients) (Tables 1 and 3). In summary, HER2-enriched cancers showed radiomic signatures that enable their separation from other breast cancers.

### Receptor status

With regard to cancer hormone receptor status, pairwise discrimination of hormone receptor-positive tumors from other groups yielded accuracies that were below 80% (see Table 2).

### External validation cohort

Of the 52 treatment-naïve, biopsy-proven breast cancers included from a second institution to validate our results, 17 were luminal A, 17 luminal B, and 18 triple negative. This allowed us to repeat some of the experiments that had yielded satisfactory results with accuracies above 80% when using our own data. Validation of these feature sets provided satisfactory results, with accuracies of 79.4% for luminal A vs. luminal B (original training dataset, 82.5%) and 77.1% for luminal B vs. triple negative (training dataset, 83.9%). Detailed results for group-wise classifications using the validation dataset can be found in Table 4, while rates of misclassified cases for pairwise comparisons (training and validation dataset) are displayed in Table 5.

**Table 4:** Results of group-wise radiomic feature-based cancer classifications (validation dataset)

	Luminal A	Luminal B	TN	All others
Luminal A	–	F, 79.4% (training, 82.5%) MI, 52.9% (training, 84.2%)	–	–
Luminal B	F, 79.4% (training, 82.5%) MI, 52.9% (training, 84.2%)	–	F, 77.1% (training, 83.9%)	F, 57.7% (training, 84.6%) POE, 51.9% (training, 89%) MI, 75% (training, 85.7%)
TN	–	F, 77.1% (training, 83.9%)	–	–
All others	–	F, 57.7% (training, 84.6%) POE, 51.9% (training, 89%) MI, 75% (training, 85.7%)	–	–

*F* Fisher, *MI* mutual information, *POE* probability of error and average correlation, *TN* triple negative

**Table 5:** Rates of misclassified cases for pairwise comparisons (training and validation dataset)

	Luminal A (*n* = 66)	Luminal B (*n* = 25)	TN (*n* = 41)	All others (*n* = 118)
Luminal A (*n* = 66)	–	Lum A vs. B: F, 9/8 (13.6/30.8%) MI, 12/13 (18.2/52%)	–	–
Luminal B (*n* = 25)	Lum A vs. B: F, 9/8 (13.6/30.8%) MI, 12/13 (18.2/52%)	–	Lum B vs. TN: F, 8/8 (32/19.5%)	Lum B vs. others: F, 17/19 (68/16.1%) POE, 17/18 (68/15.3%) MI, 12/14 (48/11.9%)
TN (*n* = 41)	–	Lum B vs. TN: F, 8/8 (32/19.5%)	–	–
All others (*n* = 118)	–	Lum B vs. others: F, 17/19 (68/16.1%) POE, 17/18 (68/15.3%) MI, 12/14 (48/11.9%)	–	–

*F* Fisher, *MI* mutual information, *POE* probability of error and average correlation, *TN* triple negative

## Discussion

In this study, we evaluated the diagnostic performance of CE-MRI coupled with radiomic analysis for the non-invasive differentiation of breast cancers with different receptor status and molecular subtypes. We hypothesized that microstructural differences between breast cancers of different receptor status, and hence molecular subtypes, would lead to different, larger-scale gray-level patterns in CE-MR images which can be assessed quantitatively by radiomic analysis. Our results demonstrate that radiomic features derived from CE-MRI data enable the determination of receptor status and molecular subtypes with a high accuracy. CE-MRI radiomic signatures may therefore provide valuable prognostic indicators based on the entire tumor and thus may be used to monitor spatio-longitudinal tumor biology changes during treatment.

In our study, the best results were achieved for the differentiation of luminal A vs. luminal B (accuracy 84.2%), luminal B vs. triple negative (83.9%), luminal B vs. all others (89%), and HER2-enriched vs. all other cancers (81.3%). In these four pairwise comparisons, especially COM and GEO features seemed to be of importance, which highlight the relevance of structural heterogeneity on the one hand and lesion shape on the other hand. External validation of part of our results yielded satisfactory results for the differentiation of luminal A vs. luminal B (79.4%) and luminal B vs. triple negative (77.1%). Our results indicate that, since tumor biology may change in course of treatment, radiomic features may possibly be able to capture these changes noninvasively, provided that our results are verified in future studies that use larger datasets and a prospective design.

Li et al. achieved excellent results for radiomic-based separation between estrogen receptor-positive and estrogen receptor-negative cancers with an area under the curve of 0.89 [17]. Given the fact that in clinical decision-making, a distinction of positivity of estrogen receptor and progesterone receptor is not relevant, we considered either estrogen receptor or progesterone receptor positivity as hormone receptor positive and did not analyze these receptors separately. In our study, in contrast to the differentiation of luminal A/B cancers, the differentiation of hormone receptor-positive and hormone receptor-negative cancers had limited success with an accuracy of 68.1%. However, we showed excellent results for the differentiation of luminal B vs. all other subtypes (89%) and luminal A vs. B cancers (84.2%). In addition to being hormone receptor positive, luminal B cancers are also characterized by other biologic features such as higher proliferation rates and/or HER2 positivity, and it seems that this can be captured through distinctive radiomic features. All hormone receptor-positive cancers are treated with endocrine therapy alone or with chemotherapy, while luminal B cancers in contrast to luminal A cancers derive benefit from additional cytotoxic and targeted treatment. In this context, the ability to identify and non-invasively spatio-longitudinally monitor these breast cancers during treatment is particularly relevant.

In this study, we used a combination of multiple radiomic features derived from different categories (e.g., COM, RLM, and ARM), all of which capture different aspects of image texture and thus might have contributed to the excellent results. This approach differs from the majority of prior MRI radiomic studies in the field of breast cancer, in which typically very few and often COM-based radiomic features are calculated [18–20, 32]. Sutton et al. achieved accuracies of up to 89.2% for the differentiation of molecular subtypes but used a combination of pathology-derived features and COM radiomic features [32]. In this study, we intentionally chose to rely solely on radiomic features from CE-MRI and to dispense with features derived from invasive tissue sampling. Holli-Helenius aimed to differentiate between luminal A and B subtypes in a small collective of 27 patients, using COM features for radiomic analysis [33]. With areas under the curve of 0.83–0.88, these results were very similar to those in our own study (84.2% accuracy); in accordance to Holli-Helenius, COM features were of importance for the differentiation between those two groups. Notably, the results of Holli-Helenius mainly relied on features extracted from pre-contrast MR images, which were not included in our study. Nevertheless, our results generally confirm that luminal A and B cancers can be differentiated by MRI-based radiomic features with a high level of confidence.

Wang et al. developed a model to exclusively distinguish triple-negative cancer from other subtypes and achieved an area under the curve of 0.88 using COM features extracted from both the tumor and healthy breast parenchyma [21]. These results agree with our findings where we demonstrated a differentiation of triple negative from luminal B with an accuracy of 83.9% when employing radiomic features from the tumor itself but not from the healthy breast tissue. The good differentiation between triple-negative and luminal B cancers may be explained by the fact that the geometric features (Table 3) used for this task capture the typically round/circumscribed shape of triple negative and the irregular/spiculated shape of luminal B cancers. In addition, Fan et al. aimed to develop a model to distinguish between molecular breast cancer subtypes based on CE-MRI data of the tumor and healthy breast parenchyma [19]. They used COM and GEO radiomic features, dynamic enhancement features, and clinical parameters and achieved good to excellent discrimination of luminal A, luminal B, HER2-enriched, and triple-negative cancers from all other tumors. Subtype discrimination in this study was partially less successful than our study (e.g., for luminal B cancers) even though we did not incorporate clinical parameters, and in part more successful (e.g., for triple-negative cancers). While it must be noted that Fan et al. used a training and a validation dataset—a strategy which, especially in combination with artificial neural networks, is preferable when datasets are sufficiently large—they did not use the same set of radiomic features in the two datasets; thus, no true validation of their model was performed.

External validation of part of our results yielded satisfactory results for the differentiation of luminal A vs. luminal B (79.4%) and luminal B vs. triple negative (77.1%). These results might be attributed to the feature sets produced by Fisher coefficients in these two comparisons, because they contained only GEO features for luminal A vs. luminal B and two GEO features and one HIS feature for luminal B vs. triple negative (see Additional file 2: Table S2). Unlike texture features, GEO and HIS feature should not be sensitive to MR image acquisition parameters. As expected, due to technical differences (TR/TE, scanner model and vendor, etc.) between training and validation datasets, some of the other results were poor, indicating a partial lack of generalizability to a wider clinical setting. Notably, some were instances where class imbalance led to seemingly good results despite a lack of separability, for instance the differentiation of luminal B vs. all others (75%). In contrast to prior CE-MRI radiomic studies that used heterogeneous image datasets in terms of acquisition parameters [11, 16–18], the MRI protocol in this study was homogeneous, which might have contributed to the excellent results. Grimm et al. evaluated associations of molecular breast cancer subtypes with imaging characteristics that included morphologic, radiomic, and dynamic enhancement features using a semi-automatic approach for lesion segmentation (i.e., a fuzzy C-Means clustering algorithm [16]. They found that enhancement characteristics and morphological features were superior to radiomic features. However, in their study, there was a high level of heterogeneity in terms of scanning equipment and pulse sequence protocols. In particular, matrix size, which, through its association with spatial resolution (and thus, pixel/voxel size), has been shown to have a major impact on feature calculations [34] and thus could be responsible for the divergent results. Similar MRI acquisition protocol heterogeneities may also have contributed to the disappointing performance of radiomic features in the study by Mazurowski et al. that investigated associations of molecular subtypes and semi-automatically extracted CE-MRI data, including COM and GEO features [11]. In contrast to prior publications that investigated molecular subtyping with radiomic analysis, a novelty and strength of the present study is our assessment of a wealth of radiomic features derived from a homogeneous state-of-the-art MRI protocol. These prior divergent results highlight the necessity for rigorous standardization across institutions, vendors, and platforms to fully explore the potential of MRI radiomics in breast cancer, especially when sample sizes are limited. On the other hand, in truly large datasets (“big data”) in which a sufficiently large number of cases for each protocol variation is available, protocol heterogeneity could present an advantage with regard to generalizability.

**Fig. 3:**
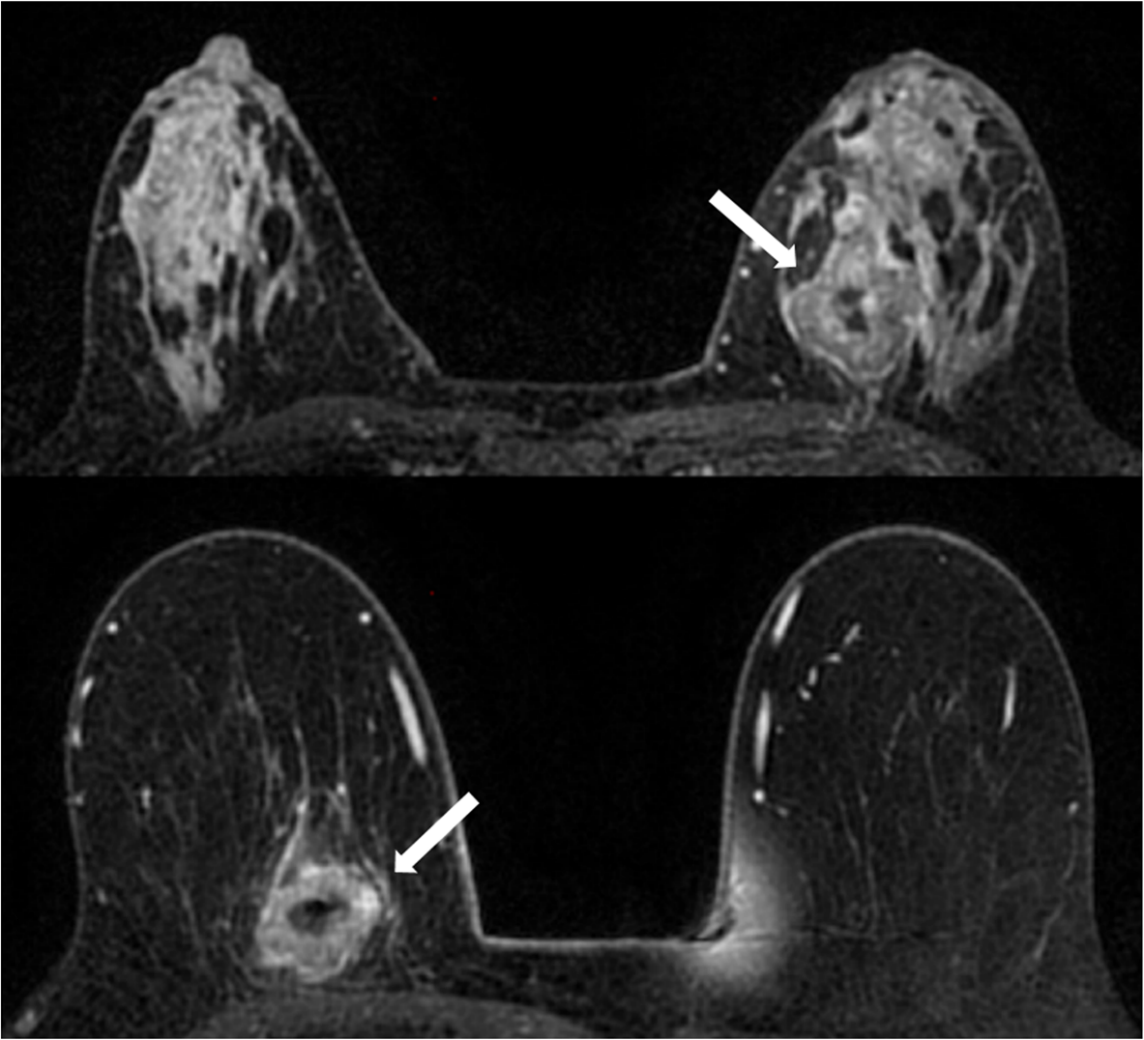
Top: contrast-enhanced fat-saturated T1-weighted image of a 50-year-old patient with a HER2-enriched cancer in the left breast. Bottom: contrast-enhanced T1-weighted image of a 59-year-old patient with a triple-negative cancer in the right breast. Both lesions are irregularly shaped and margined, with heterogeneous contrast enhancement and central necrosis. Radiomic signatures derived from contrast-enhanced MRI (CE-MRI) accurately differentiated HER2-enriched from triple-negative breast cancer with an overall accuracy of 73.5% in our patient collective

We acknowledge the limitations of the present study. In this study, molecular subtypes were derived not from formal genetic testing but from IHC surrogates. It has to be acknowledged that there is variable agreement between classifications via these surrogates and formal genetic testing (41–100%). However, in clinical practice, molecular subtypes and subsequent clinical decision-making are most often based on IHC surrogates. Like the vast majority of previous radiomic studies, the conclusions that can be drawn from this study are somewhat limited due to its retrospective nature and the unequal distribution of molecular subtypes. Another drawback is (in comparison to luminal breast cancers) the smaller number of HER2-enriched and triple-negative cancers in our patient collective (Fig. 3); however, this reflects the normal distribution of molecular subtypes of breast cancer in a patient population. Due to this limitation, we decided not to divide our original study population into a training dataset and a validation dataset. Instead, we used a *k*-nearest neighbor classifier with leave-one-out cross-validation, a technique that does not require two separate datasets and has been used in numerous studies in the field [22, 35, 36], for all pairwise comparisons, even though *n*-fold cross-validation may have produced better results in the larger samples. It has to be acknowledged that as each training set used in leave-one-out cross-validation only differs by one sample, the models tend to be more highly correlated and less variability is encountered when training the model. Thus, there is a possibility of underestimating the error via leave-one-out cross-validation. As the dataset is relatively small (especially for some subgroups such as HER2-enriched and luminal B cancers), there is the possibility that some of our findings could be the product of overfitting. Nevertheless, we aimed to decrease the risk of overfitting by reducing the dimensionality of feature space to 10% of the samples out of the original ten features provided by MaZda (e.g., five features for 50 samples). Validation of the promising results of this initial study with larger patient numbers is currently ongoing. We acknowledge that the mean size of breast cancers included in this study was relatively large, which could explain the high number of triple-negative cancers. However, this would be the target population for neoadjuvant treatment where radiomic analysis may be used to monitor spatio-longitudinal tumor biology changes during treatment and thereby identify a treatment-resistant phenotype. Unlike techniques such as 18F-fluoroestradiol positron emission tomography, which directly visualizes functional estrogen receptors, radiomics cannot directly capture receptor density. However, estrogen/progesterone receptor responsiveness “fuels” certain growth patterns, which may be captured by radiomic signatures. GEO features are influenced by tumor size to some degree; however, the texture feature classes that reflect homogeneity/heterogeneity of the lesions should not be significantly influenced by tumor size. For instance, COM features are calculated with a maximum interpixel distance of *n* = 5; since in-plane resolution was approximately 1.26 mm, feature calculation would not have been affected in tumors of 1 cm or larger. We acknowledge that a comparative analysis of images and corresponding cross-sectional histological stains would be of special interest. However, a confident co-registration is challenging, and for tumors treated with neoadjuvant chemotherapy, treatment-naïve tumor specimens are not available. Breast cancers were delineated manually and on the slice with the largest lesion diameter only. The combination of CE-MRI with diffusion-weighted imaging data might further improve the results of radiomic analysis, as different aspects of tumor biology could be captured, i.e., heterogeneity in terms of perfusion on the one hand and diffusivity/cell density on the other hand. In addition, the integration of radiomic features from healthy breast parenchyma might also be of great interest [21].

## Conclusion

In conclusion, radiomic signatures with CE-MRI may enable the assessment of breast cancer receptor status and molecular subtypes. Our initial results indicate that CE-MRI radiomic signatures may have the potential to provide prognostic indicators derived from the tumor in its entirety; theoretically, this information could be used to monitor spatio-longitudinal tumor biology changes during treatment. However, larger prospective studies and efforts in data and protocol standardization are warranted to validate these findings before definitive conclusions with regard to the value of CE-MRI radiomic features for distinguishing between breast cancer receptor status and molecular subtypes can be drawn.

## Additional files

Additional file 1: **Table S1.** Detailed results of group-wise radiomic feature-based cancer classifications for molecular breast cancer subtypes / receptor status (training dataset). The number of features used for classification (one feature per every ten sample) is written above the accuracies. The feature selection algorithm and number of most discriminating features are given in parentheses. (DOCX 24 kb)

Additional file 2: **Table S2.** Selected features sets for all pairwise classifications (training dataset). Values isn parentheses represent coordinates: information about direction and interpixel distance for pixel pairs. The full list of features and their abbreviations can be accessed at http://www.eletel.p.lodz.pl/programy/mazda/download/FeaturerList.pdf (DOCX 26 kb)

## Data Availability

The datasets used and/or analyzed during the current study are available from the corresponding author on reasonable request.

## Abbreviations

ARM: Autoregressive model
COM: Co-occurrence matrix
CE-MRI: Dynamic contrast-enhanced magnetic resonance imaging
GEO: Lesion geometry
GRA: Absolute gradient
HER2: Human epidermal growth factor receptor 2
HIS: First-order histogram
IHC: Immunohistochemical
LDA: Linear discriminant analysis
MDF: Most discriminating feature
POE + ACC: Probability of error and average correlation
RLM: Run-length matrix
WAV: Discrete Haar wavelet transform
