# Supplementary material for: Radiomic signatures with contrast-enhanced magnetic resonance imaging for the assessment of breast cancer receptor status and molecular subtypes: initial results
Source: Breast Cancer Res. 2019 Sep 12;21:106. doi: 10.1186/s13058-019-1187-z (PMC6739929; doi:10.1186/s13058-019-1187-z)
Supplement: Supplementary file 1 — Table S1. Detailed results of group-wise radiomic feature-based cancer classifications for molecular breast cancer subtypes / receptor status (training dataset). The number of features used for classification (one feature per every ten sample) is written above the accuracies. The feature selection algorithm and number of most discriminating features are given in parentheses. (DOCX 24 kb) [file 13058_2019_1187_MOESM1_ESM.docx]

**Table S1. Detailed results of group-wise radiomic feature-based cancer classifications for molecular breast cancer subtypes / receptor status (training dataset)**

|  | **Luminal A** | **Luminal B** | **HER2-enriched** | **TN** | **HR positive** | **HER2 positive** | **HR negative** | **HER2 negative** | **All others** |
| --- | --- | --- | --- | --- | --- | --- | --- | --- | --- |
| **Luminal A** | - | 6 best:  **82.5%** (F; 1)  75.4% (POE; 1)  **84.2%** (MI; 1) | 6 best:  71.7% (F; 2)  76.7% (POE; 1)  68.3% (MI; 1) | 7 best:  54.2% (F; 1)  69.4% (POE; 1)  62.5% (MI; 1) | - | - | - | - | 9 best:  55% (F; 1)  56% (POE; 1)  60.4% (MI; 1) |
| **Luminal B** | 6 best:  **82.5%** (F; 1)  75.4% (POE; 1)  **84.2%** (MI; 1) | - | 2 best:  73.7% (F; 1)  52.6% (POE; 1)  26.3% (MI; 1) | 3 best:  **83.9%** (F; 1)  76.7% (POE; 1)  54.8% (MI; 1) | - | - | - | - | 9 best:  **84.6%** (F; 1)  **89%** (POE; 1)  **85.7%** (MI; 1) |
| **HER2-enriched** | 6 best:  71.7% (F; 2)  76.7% (POE; 1)  68.3% (MI; 1) | 2 best:  73.7% (F; 1)  52.6% (POE; 1)  26.3% (MI; 1) | - | 3 best:  64.7% (F; 1)  73.5% (POE; 1)  58.8% (MI; 1) | 7 best:  79.4% (F; 1)  75% (POE; 1)  73.5% (MI) | - | - | - | 9 best:  **80.2%** (F; 1)  72.5% (POE, 1)  **81.3%** (MI; 1) |
| **TN** | 7 best:  54.2% (F; 1)  69.4% (POE; 1)  62.5% (MI; 1) | 3 best:  **83.9%** (F; 1)  76.7% (POE; 1)  54.8% (MI; 1) | 3 best:  64.7% (F; 1)  73.5% (POE; 1)  58.8% (MI; 1) | - | 8 best:  71.3% (F; 1)  70% (POE; 1)  65% (MI; 1) | 4 best:  59.5% (F; 1)  POE failed  52.4% (MI; 1) | - | - | 9 best:  70.3% (F; 1)  62.6% (POE; 1)  73.6% (MI; 1) |
| **HR positive** | - | - | 7 best:  79.4% (F; 1)  75% (POE; 1)  73.5% (MI; 1) | 8 best:  71.3% (F; 1)  70% (POE; 1)  65% (MI; 1) | - | - | 9 best:  57.1% (F; 1)  67% (POE; 1)  57.1% (MI; 1) | - | 9 best:  57.1% (F; 1)  67% (POE; 1)  57.1% (MI; 1) |
| **HER2 positive** | - | - | - | 4 best:  59.5% (F; 1)  POE failed  52.4% (MI; 1) | - | - | - | 9 best:  73.6% (F; 1)  67% (POE; 1)  69.2% (MI; 1) | 9 best:  73.6% (F; 1)  67% (POE; 1)  69.2% (MI; 1) |
| **HR negative** | - | - | - | - | 9 best:  57.1% (F; 1)  67% (POE; 1)  57.1% (MI; 1) | - | - | - | 9 best:  57.1% (F; 1)  67% (POE; 1)  57.1% (MI; 1) |
| **HER2 negative** | - | - | - | - | - | 9 best:  73.6% (F; 1)  67% (POE; 1)  69.2% (MI; 1) | - | - | 9 best:  73.6% (F; 1)  67% (POE; 1)  69.2% (MI; 1) |
| **All others** | 9 best:  55% (F; 1)  56% (POE; 1)  60.4% (MI; 1) | 9 best:  **84.6%** (F; 1)  **89%** (POE; 1)  **85.7%** (MI; 1) | 9 best:  **80.2%** (F; 1)  72.5% (POE; 1)  **81.3%** (MI; 1) | 9 best:  70.3% (F; 1)  62.6% (POE; 1)  73.6% (MI; 1) | 9 best:  57.1% (F; 1)  67% (POE; 1)  57.1% (MI; 1) | 9 best:  73.6% (F; 1)  67% (POE; 1)  69.2% (MI; 1) | 9 best:  57.1% (F; 1)  67% (POE; 1)  57.1% (MI; 1) | 9 best:  73.6% (F; 1)  67% (POE; 1)  69.2% (MI; 1) | - |

Note: F, Fisher; HER2, human epidermal growth factor receptor 2; HR, hormone receptor; MI, mutual information; POE, probability of error and average correlation; TN, triple negative.
