# Supplementary material for: Radiomic signatures with contrast-enhanced magnetic resonance imaging for the assessment of breast cancer receptor status and molecular subtypes: initial results
Source: Breast Cancer Res. 2019 Sep 12;21:106. doi: 10.1186/s13058-019-1187-z (PMC6739929; doi:10.1186/s13058-019-1187-z)
Supplement: Supplementary file 2 — Table S2. Selected features sets for all pairwise classifications (training dataset). Values isn parentheses represent coordinates: information about direction and interpixel distance for pixel pairs. The full list of features and their abbreviations can be accessed at http://www.eletel.p.lodz.pl/programy/mazda/download/FeaturerList.pdf (DOCX 26 kb) [file 13058_2019_1187_MOESM2_ESM.docx]

**Table S2. Selected features sets for all pairwise classifications (training dataset)**

| **Luminal A vs Luminal B** | | | | | **Luminal A vs HER2-enriched** | | | | | **Luminal A vs TN** | | | | **Luminal B vs HER2-enriched** | | | | | **Luminal B vs TN** | | |
| --- | --- | --- | --- | --- | --- | --- | --- | --- | --- | --- | --- | --- | --- | --- | --- | --- | --- | --- | --- | --- | --- |
| **Fisher** | **POE** | | **MI** | | **Fisher** | **POE** | | **MI** | | **Fisher** | **POE** | | **MI** | **Fisher** | **POE** | | **MI** | | **Fisher** | **POE** | **MI** |
| GeoW7  GeoXYo  GeoXo  GeoSigR  GeoW9  GeoW3 | S(4,4)Correlat  Teta1  S(1,-1)SumOfSqs  S(1,1)SumOfSqs  GrKurtosis  Teta4 | | S(3,3)SumAverg  S(2,2)SumEntrp  S(5,-5)DifEntrp  45dgr_LngREmph  S(2,-2)SumAverg  S(0,1)Contrast | | GeoU1  GeoN1  GeoLminE  GeoNc  GeoUg  GeoRc2 | S(0,3)SumOfSqs  S(5,5)Correlat  Perc.10%  S(5,0)DifVarnc  S(3,3)InvDfMom  Teta4 | | Teta2  S(5,-5)SumVarnc  S(1,0)SumVarnc  S(0,5)DifVarnc  GeoRm  WavEnHL_s-1 | | S(0,1)AngScMom  S(0,1)Entropy  S(0,2)Entropy  S(0,3)Entropy  S(1,-1)Entropy  S(0,4)AngScMom  S(0,2)AngScMom | S(5,-5)SumOfSqs  Teta2  GeoRff  GeoRd  Variance  S(1,1)AngScMom  S4,-4)SumOfSqs | | GeoRff  GrVariance  S(0,3)SumEntrp  S(3,3)Entropy  S(2,-2)SumEntrp  S(5,-5)Entropy  S(5,0)Entropy | S(3,3)InvDfMom  S(5,5)InvDfMom | Teta3  GrSkewness | | S(2,-2)DifEntrp  S(2,0)SumVarnc | | GeoW7  Variance  GeoW9 | Kurtosis  S(4,4)DifEntrp  Teta1 | GeoM2y  S(5,-5)DifEntrp  S(1,1)SumOfSqs |
| **HER2-enriched vs TN** | | | | | **HER2-enriched vs HR positive** | | | | | **TN vs HR positive** | | | | **TN vs HER2 positive** | | | | | **HR positive vs HR negative** | | |
| **Fisher** | | **POE** | | **MI** | **Fisher** | | **POE** | | **MI** | **Fisher** | | **POE** | **MI** | **Fisher** | **POE** | | | **MI** | **Fisher** | **POE** | **MI** |
| WavEnLH_s-4  S(5,5)InvDfMom  S(3,3)InvDfMom | | S(2,0)AngScMom  S(5,5)SumOfSqs  Perc.10% | | S(2,-2)SumEntrp  S(4,-4)SumEntrp  S(4,0)InvDfMom | S(5,5)InvDfMom  Horzl_GLevNonU  135dr_GLevNonU  45dgr_GLevNonU  Vertl_GLevNonU  Horzl_RLNonUni  GeoF | | S(3,-3)SumOfSqs  GeoXYo  Perc.10%  Teta4  S(3,3)InvDfMom  S(5,0)DifVarnc  S(1,1)AngScMom | | Vertl_Fraction  S(0,1)InvDfMom  Teta2  S(3,-3)SumEntrp  S(5,5)SumAverg  S(4,-4)SumEntrp  GeoE1 | S(0,1)AngScMom  S(0,1)Entropy  GeoAox  S(0,4)AngScMom  S(0,3)Entropy  S(0,2)Entropy  S(0,3)AngScMom  S(0,2)AngScMom | | S(5,-5)SumOfSqs  GeoY  GeoM2y  Variance  Teta2  S(3,-3)DifVarnc  S(4,-4)SumOfSqs  S(1,-1)SumEntrp | GeoM2y  S(0,3)SumEntrp  S(4,0)SumVarnc  S(1,1)AngScMom  S(4,4)SumEntrp  GeoFv  GeoY  S(5,-5)Entropy | GeoW9  GeoW3  S(1,-1)SumAverg  GeoRs | | POE failed | GeoRm  GeoRs  S(2,-2)SumEntrp  S(1,-1)SumEntrp | | S(0,1)AngScMom  S(1,1)SumEntrp  S(0,1)Entropy  S(1,-1)Entropy  S(0,2)Entropy  S(2,-2)Entropy  S(0,2)AngScMom  S(1,-1)AngScMom  S(0,1)SumEntrp | WavEnHH_s-1  S(0,5)SumOfSqs  GeoRd  GeoXo  Teta4  GeoRff  S(0,1)AngScMom  Perc.50%  WavEnHL_s-3 | S(4,0)SumVarnc  GeoM2y  GeoW4  S(1,1)SumEntrp  S(1,1)AngScMom  S(5,0)SumVarnc  S(1,0)AngScMom  GeoRff  S(4,0)SumOfSqs |
| **HER2 positive vs HER2 negative** | | | | | **Luminal A vs all others** | | | | | **Luminal B vs all others** | | | | **HER2-enriched vs all others** | | | | | **TN vs all others** | | |
| **Fisher** | | **POE** | | **MI** | **Fisher** | | **POE** | | **MI** | **Fisher** | | **POE** | **MI** | **Fisher** | | **POE** | **MI** | | **Fisher** | **POE** | **MI** |
| GeoW3  GeoEr2  GeoSigR  GeoW9  GeoM2y  GeoFE  GeoFd2  GeoU1  GeoN1 | | S(0,4)SumOfSqs  Teta1  GeoW11  S(5,0)SumAverg  Perc.10%  S(5,0)InvDfMom  Teta4  GeoSigR  WavEnHL_s.3 | | GeoRs  S(4,4)DifEntrp  S(1,1)DifVarnc  S(1,0)DifVarnc  GeoRff  S(5,-5)Contrast  S(0,2)Contrast  S(5,-5)DifEntrp  S(0,19Contrast | S(0,1)AngScMom  S(0,1)Entropy  S(1,-1)Entropy  S(0,2)Entropy  S(0,2)AngScMom  S(1,0)Entropy  S(1,1)AngScMom  S(1,0)AngScMom  S(1,-1)AngScMom | | GeoXo  GeoRd  S(5,-5)SumOfSqs  Teta1  GeoRff  GeoYo  S(0,4)SumOfSqs  GeoAox  Perc.50% | | GeoRff  S(1,-1)Entropy  S(2,-2)SumOfSqs  S(0,1)AngScMom  S(4,0)Entropy  S(0,1)Entropy  S(2,0)SumEntrp  S(1,1)SumEntrp  S(1,0)DifEntrp | GeoW7  GeoW9  GeoXYo  GeoM2y  GeoXo  GeoRs  GeoW3  GeoSigR  GeoNo | | S(5,5)InvDfMom  Teta1  S(1,1)SumOfSqs  S(1,-1)SumOfSqs  S(2,2)SumAverg  GrKurtosis  Teta4  S(1,0)SumOfSqs  S(4,4)InvDfMom | S(1,1)SumOfSqs  GeoMaver  S(3,3)SumEntrp  S(0,1)DifEntrp  S(1,0)InvDfMom  S(1,-1)SumOfSqs  GeoY  S(1,0)DifVarnc  S(3,0)InvDfMom | S(5,5)nvDfMom  GeoRd  S(0,5)InvDfMom  GeoU1  GeoN1  GeoSxL  S(4,-4)InvDfMom  Horzl_RLNonUni  GeoF | | Teta4  S(5,5)InvDfMom  S(5,0)DifEntrp  GeoXYo  Teta2  S(4,0)SumOfSqs  S(3,3)InvDfMom  S(5,0)DifVarnc  GeoY | S(0,1)InvDfMom  S(5,5)SumAverg  S(1,1)Contrast  S(2,-2)Contrast  GeoE12  Vertl_ShrtREmp  S(2,-2)DifEntrp  S(0,2)Entropy  S(4,-4)SumEntrp | | S(0,1)AngScMom  S(0,1)Entropy  GeoAox  (0,4)AngScMom  S(1,-1)SumAverg  S(0,3)AngScMom  S(0,2)AngScMom  S(2,-2)SumAverg  S(0,5)AngScMom | S(5,-5)SumOfSqs  GeoY  GeoM2y  Teta2  Variance  S(1,-1)SumEntrp  S(3,-3)DifVarnc  GeoAox  S(5,5)SumOfSqs | S(1,1)AngScMom  GeoM2y  S(0,3)SumEntrp  S(5,0)SumVarnc  S(4,0)DifEntrp  S(4,4)SumEntrp  GeoW7  S(4,0)SumVarnc  S(0,1)SumAverg |

Note: HER2, human epidermal growth factor receptor 2; HR, hormone receptor; MI, mutual information; POE, probability of error and average correlation; TN, triple negative.
